# Information Diet in Covid-19 Crisis; a Commentary

**Published:** 2020-03-22

**Authors:** Hasan Ashrafi-rizi, Zahra Kazempour

**Affiliations:** 1 Associate Professor, Medical Library and Information Science Department, Health Information Technology Research Center, Isfahan University of Medical Sciences, Isfahan, Iran; 2 Library and Information Science Department, Faculty of Media, Payame Noor University, Tehran, Iran

**Keywords:** Coronavirus, Information Diet, Information Obesity, Disaster Planning, Confidential Information

## Introduction

By the beginning of COVID-19 crisis in China in late 2019, and its spread throughout the world in early 2020, countries around the world experienced numerous problems (1).The outbreak of the new Coronavirus started in Wuhan, and this third epidemic of Coronaviruses expanded to the middle east promptly. . Therefore, the World Health Organization (WHO) expressed its concerns about the Coronavirus crisis (2). This crisis caused production and publication of large amounts of valid and invalid information, eventually leading to information obesity phenomenon. Information obesity can have many negative consequences on the general population, causing major problems for governments, especially if the amount of invalid information is too large. It is worth to mention that however almost impossible, controlling and monitoring media is a massive challenge for different governments. Hence, individuals should protect themselves against unreliable information, and pursue an authentic “information diet”. In the present study, authors have explained and interpreted the concept of the information diet, proposed by Johnson, based on scientific evidence, observation of media news and the social media environment, to help maintain the use of valid information in facing the new Coronavirus crisis.

## Information Diet in crisis condition

Due to the large amount of information production and publication during the current Coronavirus crisis, serious concerns have been raised over people's excessive information consuming. “If information consumption grows, it can cause a loss of tranquility, confusion and can concern the human mind, rather than being a facilitator of life. Therefore, as humans respect the limits about use of water, food, and air, they must also rationally limit their consumption of information; otherwise they will encounter phenomena known as the information obesity, and the information diet will be a rational solution to this phenomenon” (3). The concept of Information Diet was introduced by Clay Johnson for the first time. He believes consumers should take responsibility for the type of information they consume, likewise the rational consumption of food. He also argues that there must be a sustainable news movement, like a sustainable food movement that has already started in the US. In other words, people and even governments are not capable of dealing with the media to a great extent, but people can create their own information diets, like food diet. Therefore, information consumers are advised to consume information in a limited way. However, they must demand complete and credible news, not news that is based on emotional content and full of advertisements” (4). In the present Coronavirus crisis, some people spend most of their time studying related information regarding Covid-19 in written and virtual media; Meanwhile, they are unwilling to spend time exercising at home, talking to their families, playing with their children or doing other useful tasks such as studying, watching movies and so on. To seriously address these problems in the Coronavirus crisis, not only people must have optimum consumption of appropriate information, but also consuming information should comply with specific terms and conditions. For example, children should not be exposed to the news related to the Coronavirus, because this information increases anxiety and causes other psychological problems among them. The elderly is also vulnerable to the spread of information, and should be provided with reliable and limited information, and they should not be given the ongoing news. On the other hand, individuals should be able to distinguish between high quality and poor quality information, since much of the information regarding Covid-19 is invalid and is produced and published for economic, political, social, and in general profitable purposes (disinformation). Nowadays, information is being provided every day, and by contemplating on and reviewing the publication of statistics related to the Coronavirus crisis on websites, blogs, social media, television and satellite channels etc., it can be understood that the overload of information has already occurred. Hence, providing people with all kinds of health literature is the only option. Although people may not have the ability to control the production and supply of the information nowadays, each of them must control the extent and type of the information which they consume. Therefore, in order to achieve a comprehensive information diet in societies, people need to be equipped with the knowledge and skills of health information literacy, media health literacy, media literacy and health literacy. Currently these trainings can only be delivered through the mass media. Delivering these skills requires specific knowledge and skills which health information professionals (medical librarians) poses, and they can train other people in the society through mass media relying on their abilities.

## Conclusion

Governments need to adopt appropriate policies and plans for managing public opinions in the times of crisis, such as the new Coronavirus crisis. The acquisition of "information evaluating skills" by people will be a prerequisite for adhering to the correct information diet, and recommending the information diet as one of the essential strategies in reducing the level of public health anxiety should be considered by health practitioners in countries such as Iran. It should be noted that anxiety and confusion, as well as an increase in people's physical and mental illnesses, including health anxieties and sometimes cyberchondria, are among the negative consequences of not adhering to information diet and consuming more than needed health information. In cyberchondria, an individual searches for a great deal of information, because of inconsistent information and uncertainty about its validity , which in turn will increase one's anxiety and stress and causes information obesity (5). This phenomenon would be one of the negative consequences of not adhering to the information diet in the current Coronavirus crisis. In general, "health media literacy" and public education through mass media are the best options for controlling and monitoring people's information exposure in the current crisis. Certainly, all of the countries need tranquility after overcoming the Coronavirus crisis, which will be achieved in the light of people's adherence to the information diet during and after the crisis.
